# Supplementary material for: A Web-Based Application to Monitor and Inform about the COVID-19 Outbreak in Italy: The {COVID-19ita} Initiative
Source: Healthcare (Basel). 2022 Mar 3;10(3):473. doi: 10.3390/healthcare10030473 (PMC8948929; doi:10.3390/healthcare10030473)
Supplement: Supplementary file 1 [file healthcare-10-00473-s001.zip › healthcare-1601605-supplementary.pdf]

healthcare

Supplementary Materials

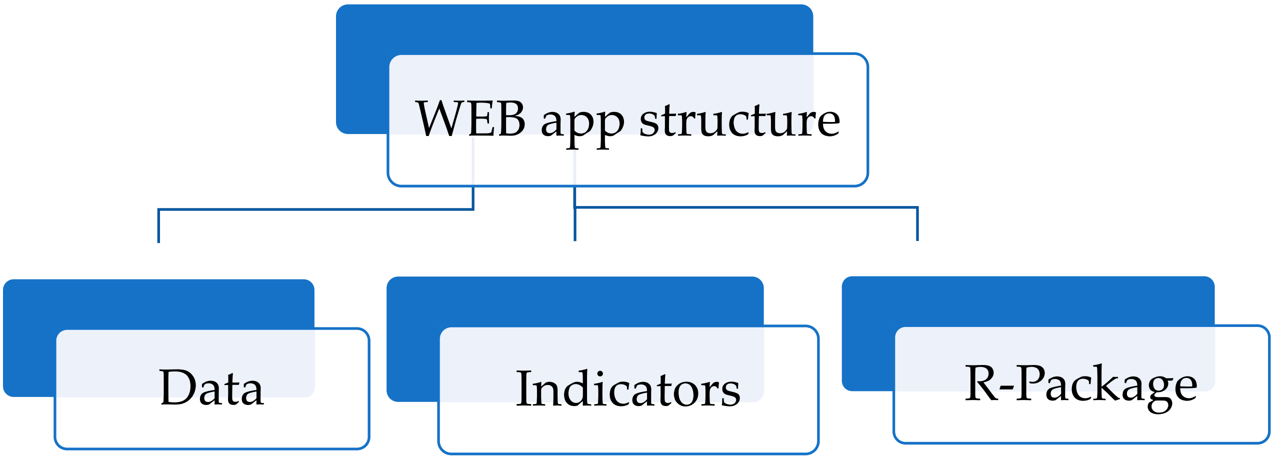

Figure S1. Web app structure handling the Covid-19 official data, the epidemic indicators, and the COVID-19ita R package providing the implementation of the web application together with a directly usable R version of the official data.

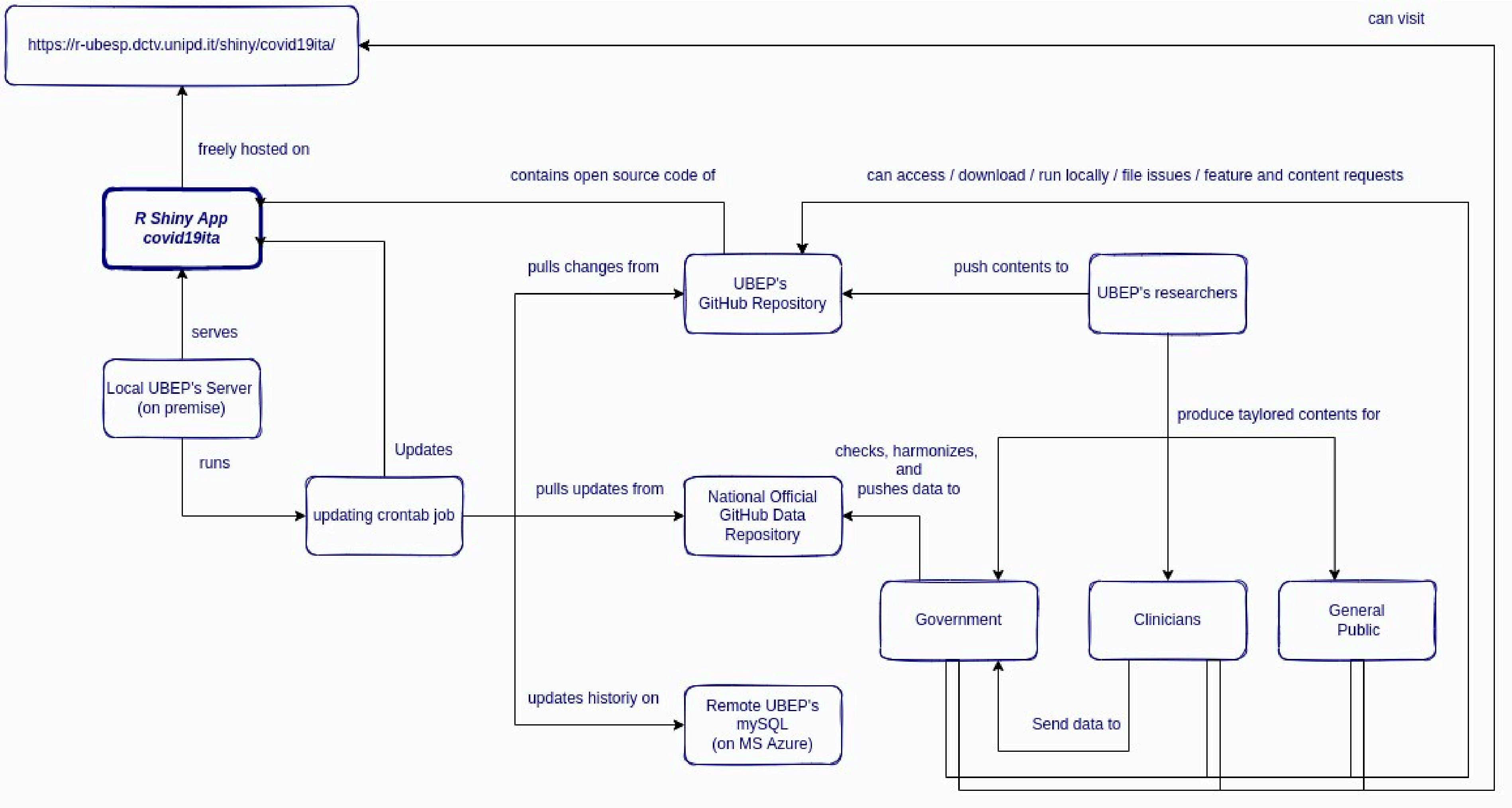

Figure S2. WEB app structure concerning relations with R code, data, environment and storing systems.
